# The progression rate of peripheral arterial disease in patients with intermittent claudication: a systematic review

**DOI:** 10.1186/s13047-019-0351-0

**Published:** 2019-08-06

**Authors:** A. Mizzi, K. Cassar, C. Bowen, C. Formosa

**Affiliations:** 1 Faculty of Health Sciences, University of Malta, Msida, Malta; 2 Mater Dei hospital, Faculty of Medicine and Surgery, University of Malta, Msida, Malta; 3 School of Health Sciences, Faculty of Environmental and Life Sciences, University of Southampton, Southampton, UK

**Keywords:** Intermittent claudication, Peripheral arterial disease, Haemodynamic deterioration, Prognosis, Progression

## Abstract

**Background:**

Intermittent claudication (IC) is the most common symptom of peripheral arterial disease and is generally treated conservatively due to limited prognostic evidence to support early revascularisation in the individual patient. This approach may lead to the possible loss of opportunity of early revascularisation in patients who are more likely to deteriorate to critical limb ischaemia. The aim of this review is to evaluate the available literature related to the progression rate of symptomatic peripheral arterial disease.

**Methods:**

We conducted a systematic review of the literature in PubMed and MEDLINE, Cochrane library, Elsevier, Web of Science, CINAHL and Opengrey using relevant search terms to identify the progression rate of peripheral arterial disease in patients with claudication. Outcomes of interest were progression rate in terms of haemodynamic measurement and time to development of adverse outcomes. Two independent reviewers determined study eligibility and extracted descriptive, methodologic, and outcome data. Quality of evidence was evaluated using the Cochrane recommendations for assessing risk of bias and was reported according to the Preferred Reporting Items for Systematic Reviews and Meta-Analyses guidelines.

**Results:**

Seven prospective cohort studies and one retrospective cohort study were identified and included in this review with the number of participants in each study ranging from 38 to 1244. Progression rate reports varied from a yearly decrease of 0.01 in ankle-brachial pressure index (ABPI) to a yearly decrease ABPI of 0.014 in 21% of participants. Quality of evidence ranged from low to moderate mostly due to limited allocation concealment at recruitment and survival selection bias.

**Conclusions:**

Progression of PAD in IC patients is probably underestimated in the literature due to study design issues. Predicting which patients with claudication are likely to deteriorate to critical limb ischaemia is difficult since there is a lack of evidence related to lower limb prognosis. Further research is required to enable early identification of patients at high risk of progressing to critical ischaemia and appropriate early revascularisation to reduce lower limb morbidity.

## Background

Intermittent claudication (IC) is the first symptom of peripheral arterial disease (PAD) and is associated with significant functional impairment [1, 2]. Patients with IC are at significant risk of atherosclerotic morbidity such as stroke and coronary artery disease [3]. The mortality risk of patients presenting with IC is double that of patients with PAD who are asymptomatic [4]. On the other hand, the prognosis and progression of PAD of the affected limbs is known to be less relevant with the majority remaining stable, some improving, while approximately 20–25% requiring revascularisation and 5% eventually deteriorating to critical limb ischaemia (CLI) [5].

Since it is expected that the majority of patients with IC will have a relatively benign lower limb prognosis, the recommended first-line treatment strategy is conservative treatment [6]. This includes lifestyle modification (smoking cessation and exercise [7, 8], and medical therapy [9–11] (antiplatelets, lipid lowering drugs and blood pressure management). The primary objective of this treatment strategy is to reduce the risk of major adverse cardiovascular events in this patient cohort rather than to control lower limb symptoms or delay progression of peripheral arterial disease. Unfortunately, this approach ignores the fact that a proportion of patients with claudication will deteriorate to critical limb ischaemia and will require lower limb interventions. Patients who develop a major adverse limb event (MALE) have more than a threefold increase in mortality and an almost two hundred fold increased risk of limb loss [12].

If those patients with claudication at high risk for deterioration to critical limb ischaemia could be identified before the onset of gangrene and tissue loss, early revascularisation could possibly reduce the risk of minor amputations, major amputations, local and systemic sepsis from ulcers and wet gangrene and mortality [13]. Intervening early when the patient is younger and possibly fitter and without concomitant ulceration or gangrene would more likely lead to better surgical outcomes, lower mortality and less septic complications. There is clear evidence that revascularisation surgery conducted on an urgent or emergency basis and in the presence of gangrene and tissue loss is associated with significantly higher surgical mortality and morbidity [13]. In addition, intervening earlier would often require less invasive and less extensive procedures [14]. Endovascular, open or hybrid procedures involve the treatment of underperfused segments in the lower limb by improving blood flow to increase pain free walking distance. While revascularisation, together with exercise, is superior in treating IC compared to medical therapy alone [15–17], the choice of treatment should rely on patients’ values and preferences, clinical context and expertise [18] since well-defined clinical practice guidelines for choice of treatment in patients with IC are still lacking.

Currently there are no predictive formulae that allow the clinician to estimate the level of risk of an individual patient with intermittent claudication to progress to critical limb ischaemia or the time scale in which this is likely to occur [6]. For effective patient specific decisions to be made, tools to predict the risk per year of the patient deteriorating to critical ischaemia are required. This risk could then be balanced against the life expectancy of the particular patient, as well as the risks of the particular intervention/s required to optimise perfusion to the limb. Availability of this information would also enable informed decisions as to which treatment option would be best suited for a particular patient where more than one treatment option is available. Thus for example a lower risk but less effective revascularisation option may be selected in a high risk patient, while for a low risk patient a higher risk but more durable procedure may be indicated.

In order to develop predictive formulae for patient specific lower limb management for IC, detailed PAD progression data is crucial, however this is scarce, since the main focus of research has been coronary disease and stroke with less attention paid to the lower limb [3, 5].

This paper evaluated the current evidence related to the progression rate of PAD in patients with IC which is essential for informed clinical decision making.

## Methods

This systematic review was conducted following recommendations from the Cochrane Collaboration [19]. The study design, population selection and follow-up time frame were summarised following the Preferred Reporting Items for Systematic Reviews and Meta-Analyses (PRISMA) guidelines [20]. The search was conducted between 9th July and 25th July 2018.

### Literature search

The search for potentially relevant articles was performed in PubMed and MEDLINE, Cochrane database of systematic reviews, Elsevier (Embase and Sciencedirect), Web of Science and CINAHL. Reference lists of retrieved full-text articles were also cross-checked and OpenGrey database was searched for any relevant grey literature. The searches were performed without restrictions on publication date, or publication status. Search results were downloaded into a bibliographic software Refworks (ProQuest LLC).

The inclusion criteria for the search strategy consisted of studies on humans and written in the English language. The search terms used for this literature search were identified after reading several publications related to the subject area and through conducting scoping searches. The terms were formulated by three experienced reviewers who have an interest in the subject area. Choice of terms was done independently and was finalised by the main researcher who eliminated duplicates but retained all the identified key words. The literature search sought to identify studies reporting the progression of PAD in patients with IC. Search terms included free text terms and Medical Subject Heading (MeSH) terms related to [1] intermittent claudication; [2] PAD; [3] peripheral vascular disease. The keywords and MeSH headings for searching MEDLINE used are presented in Table 1. Search strategies were adapted for searching within different databases. The terms needed to present in the title or abstract.

**Table 1:** Keywords and MeSH headings used for literature search

Search 1: MeSH headings – intermittent claudication Intermittent claudication AND Prognosis Intermittent claudication AND fate Intermittent claudication AND natural history Intermittent claudication AND progression Intermittent claudication AND outcome Search 2: MeSH headings - peripheral arterial disease, peripheral vascular disease Peripheral arterial disease/ peripheral vascular disease AND Prognosis Peripheral arterial disease/ peripheral vascular disease AND Progression Peripheral arterial disease/ peripheral vascular disease AND natural history Peripheral arterial disease/ peripheral vascular disease AND outcome	

### Study eligibility criteria

As recommended in the PRISMA statement [20], before starting the literature search explicit declarations of questions being addressed were defined with reference to participants, interventions, comparisons, outcomes and study design (PICOS).

Eligible articles needed to report on the natural history of patients with IC as a symptom of PAD, also documenting progression rate of the disease. Disease progression has been previously suggested to be detectable after twelve months [21], therefore studies were selected if they primarily aimed to investigate the progression of symptomatic arterial disease with at least one-year follow-up.

Primary endpoints were progression rate in terms of haemodynamic parameters (expressed as time for change in ankle and / or toe pressures) and adverse lower limb events (expressed as time to development of ulceration, amputation or gangrene). Secondary endpoints were identification of prognostic factors for the development of adverse lower limb events and for the progression of PAD in patients with IC.

While prospective observational longitudinal cohort studies have the most suitable design to investigate the natural history of events [22], in this review all study designs and sample sizes were considered.

### Study selection

Titles and abstracts of studies identified by the search strategy were assessed in terms of relevance to the study topic. Additional relevant references identified from the bibliography of the reviewed articles and those retrieved from the grey literature search, were also assessed. Full texts of selected articles were retrieved if they fulfilled the inclusion criteria and were reviewed by two investigators independently (SM and CF, both experienced researchers). A meta-analysis was planned if clinical homogeneity was observed. The process was pilot-tested on a selection of studies and refined where required. Disagreement between reviewers regarding the article relevance, inclusion or quality was discussed until agreement was reached.

### Quality assessment

Methodological quality of each trial was evaluated systematically with the aid of the Cochrane handbook [19] and reported following the PRISMA checklist [20]. The Cochrane recommended approach for interpretation of the risk of bias for each important outcome (across domains) within and across studies was applied as summarized in Table 2 below [23].

**Table 2:** Interpretation of bias risk [23]

Risk of bias	Interpretation	Within a study	Across studies
Low risk of bias.	Plausible bias unlikely to seriously alter the results.	Low risk of bias for all key domains.	Most information is from studies at low risk of bias.
Unclear risk of bias.	Plausible bias that raises some doubt about the results.	Unclear risk of bias for one or more key domains.	Most information is from studies at low or unclear risk of bias.
High risk of bias.	Plausible bias that seriously weakens confidence in the results.	High risk of bias for one or more key domains.	The proportion of information from studies at high risk of bias is sufficient to affect the interpretation of results.

## Results

**Fig. 1:**
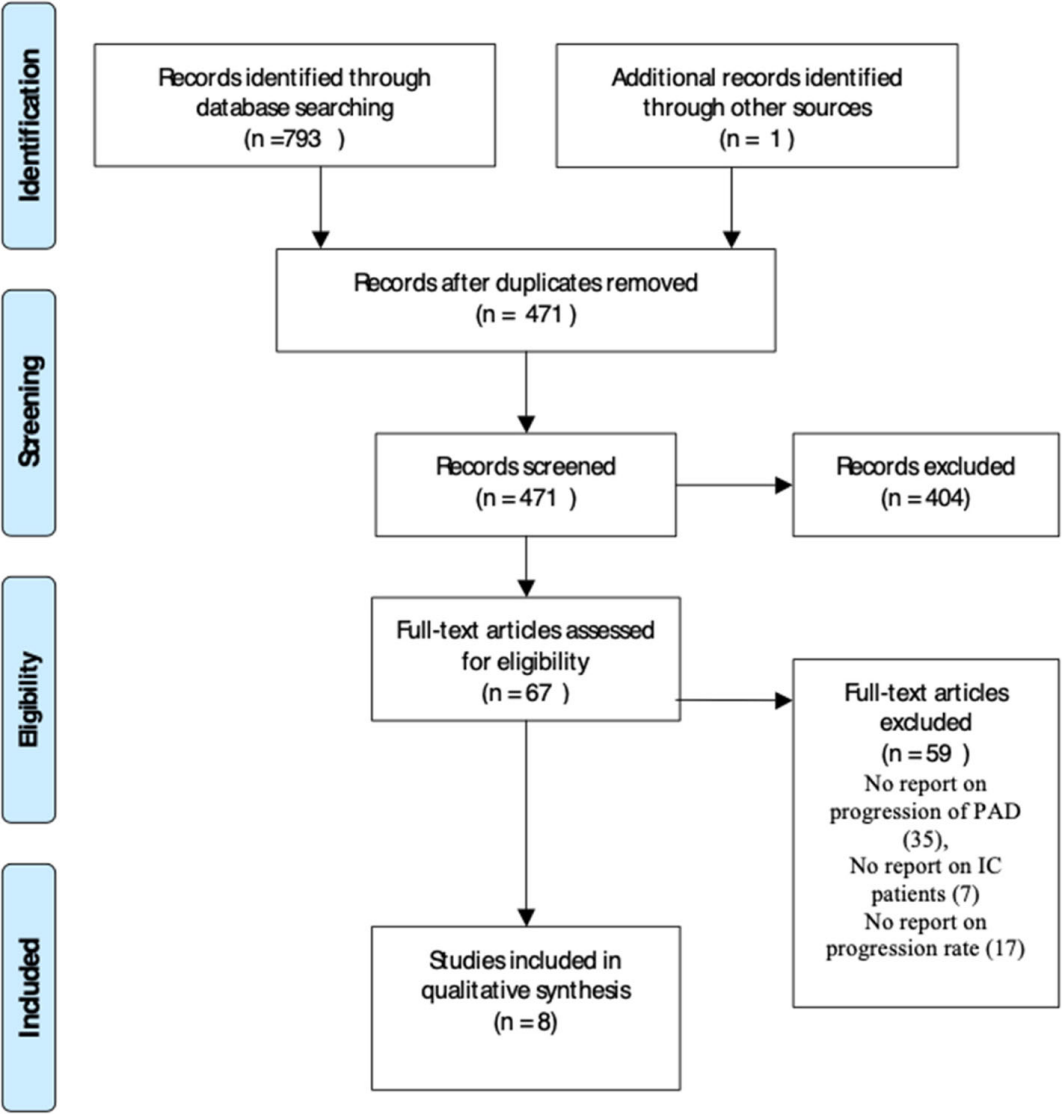
PRISMA flow chart for study selection

The initial database search yielded a total of 793 potentially relevant papers and an additional paper was retrieved from grey literature search. These were processed as illustrated in Fig. 1 and following the independent review of the full-text versions by SM and CF, a further 59 articles were excluded while 8 studies fulfilled the inclusion criteria and were included. The reasons for exclusion were failure to report haemodynamic deterioration, failure to report outcomes of participants with IC, failure to report temporal progression of PAD and use of same cohort. The PRISMA flow chart and reasons for exclusion are shown in Fig. 1. Due to the heterogeneity of the methods used in reporting haemodynamic deterioration and outcome measures, a narrative synthesis of the 8 included studies was conducted without meta-analysis.

Eight full-text articles met the selection criteria reporting temporal progression of PAD in patients with IC [21, 24–30]. Study designs included were seven prospective cohort studies and one retrospective cohort study. The number of participants ranged from 38 to 1244, with the largest study recruiting only male participants. Six studies [24–29] included ABPI as a baseline clinical measure of PAD and two studies [21, 30] reported degrees of stenosis using duplex ultrasonography. Only one study [26] included TBPI. Diagnosis of IC as a symptom of PAD, in order to exclude any alternative diagnosis, varied considerably across the studies. One study used the WHO questionnaire, while 5 studies used ABPI < 0.9 as a cut-off point and another two studies used duplex ultrasound scan reports to diagnose PAD at baseline and exclude alternative non-atherosclerotic causes of IC. Two studies evaluated walking distance using treadmill tests, one other study included data using the San Diego claudication questionnaire while the rest did not report walking distance. Follow-up period ranged from one year to twelve years. The characteristics of the included studies are described in Table 3.

**Table 3:** Characteristics of included trials

Publications	Trial characteristics	Haemodynamic measure	Progression of PAD	Prognostic factors for progression of PAD
Smith et al. 1998 [29]	Prospective observational cohort study 235 participants with IC 3 year follow-up	ABPI	ABPI decreased by > 0.14 after 1 year in 21% of participants, 12.5% developed CLI after 3 years	Hypertriglycerideamia, ABPI < 0.5 at baseline is associated with increased risk of deterioration
Smith et al. 2003 [28]	Prospective observational cohort study 131 participants with IC 5 and 12 year follow-up	ABPI	Overall decrease in ABI by 0.09 over 5 yrs. in higher ABPI limb. Decrease in ABPI by 0.04 in lower ABPI limb	Higher ABI associated with faster deterioration Lower ABI associated with increased risk of lower limb events
Walsh et al. 1991 [21]	Prospective observational cohort study 38 participants (45 limbs) with IC and SFA stenosis 3 year follow-up	Arteriograms, and duplex scans	15.6% of participants developed a 30% increase in stenosis after 1 year.	Smoking and symptom progression predictive of SFA stenosis progression. SFA occlusion is synchronous with symptom progression. Contralateral SFA occlusion increases risk of faster stenosis
Bird et al. 1999 [26]	Prospective observational cohort study 177 participants with IC 4.7 year follow-up	ABPI, San Diego Claudication questionnaire	Overall ABI decrease of 0.02 over 4.6 yrs. and TBPI decrease by 0.013 in 4.7 years	Age, DM are associated with rapid progression
Aquino et al. 2001 [24]	Prospective observational cohort study 1244 male veterans with IC 3.7 year follow-up	ABPI	Overall yearly decline in ABPI by 0.014	ABPI < 0.5, high smoking pack years and DM increase risk CLI
Naschitz et al. 1988 [27]	Retrospective cohort study 460 participants with IC referred for vascular surgical consultation 3.8 year follow-up	ABPI, Doppler waveforms, angiography	Decrease by 0.15 in ABPI in those who deteriorate (53.2% of participants)	ABI > 0.7, no deterioration of ABI predicts good outcome ABI < 0.5 at baseline increases risk of deterioration by 3.8 times ABI decrease by 0.15 increases risk by 1.9 times for need eventual surgery
Fowkes et al. 1993 [25]	Prospective observational cohort study 617 participants with IC 1 year follow-up	ABPI	Overall ABPI decrease by -- 0.01 per year 4.8% developed CLI, no haemodynamic report	Age, smoking associated with deterioration in ABPI
Whyman et al. 1993 [30]	Prospective observational cohort study 38 participants with IC with femoropopliteal artery stenosis 19 months	Duplex scan (Bollinger score)	Patients with velocity ratio > 3 progressed to occlusion within 13 weeks	Velocity ratio of > 3 associated with occlusion within 76 weeks.

### Progression of PAD

We identified 8 studies which evaluated the progression of PAD in patients with IC. Only two studies [24, 25] reported yearly haemodynamic decline in ABPI by 0.014 and 0.01 respectively. Others reported varied results which are summarized below. The different measures of PAD progression, differing follow-up and outcome data used in the included literature makes inferences difficult as to defining the progression rate of PAD in patients with IC.

### Summary of results

In summary, the results of this study demonstrate that yearly haemodynamic decline in ABPI was reported in two study by 0.014 [24] and 0.01 [25]. Another study reported an overall decline in ABPI of 0.02 or 0.013 in TBPI, but included both IC participants and participants with PAD but with no symptoms [26]. Others reported that ABPI < 0.58 at baseline and/or a decline by 0.15 in ABPI is indicative of progression to critical limb ischaemia (CLI), without reporting the temporal element [27]. However, a faster atherosclerotic progression rate was observed in claudicants compared to non-claudicants [21] and a yearly 30% increase in SFA stenosis is indicative of requirement for revascularisation. Smith et al. [28] reported a decline after 5 years in ABPI by 0.04 in the limb with lower ABPI at baseline and a decline of 0.09 in the limb with higher ABPI. While in an earlier study, Smith et al. [29] reported a decline of 0.14 of ABPI in 21% of the participants after 1 year of diagnosis. Using velocity ratio measured by duplex, Whyman et al. [30] reported that a velocity ratio > 3 at baseline is associated with deterioration to occlusion of SFA within thirteen weeks.

The prognostic value of the reports presented in the selected studies is limited due to the quality of evidence of each trial. Overall the reviewers rated the quality of evidence for progression rate of PAD in patients with IC as low mainly owing to the possibility of serious risk of bias in these studies (Table 4). For evidence related to the identification of specific patient characteristics associated with poor prognosis and risk of developing adverse events, reviewers generally rated the evidence as moderate, mainly due to the substantial differences between the studies. A narrative with the rationale for these results is presented below.

**Table 4:** Risk of bias of included trials

Source of bias					
First author	Allocation concealment	Selective recruitment	Incomplete outcome data	Survival selection	Summary risk of bias
Naschitz 1988 [27]	yes	yes	no	yes	high
Walsh (1991) [21]	yes	yes	no	no	moderate
Fowkes (1993) [25]	yes	yes	no	yes	high
Whyman (1993) [30]	No	No	yes	No	low
Bird (1999) [26]	yes	yes	yes	yes	High
Smith (1998) [29]	No	yes	yes	yes	high
Smith (2003) [28]	No	yes	yes	yes	high
Aquino (2005) [24]	no	yes	no	yes	moderate

Selection bias, because of inadequate allocation concealment at recruitment stage was observed in four out of the seven studies reviewed [21, 25–27]. Inadequate allocation concealment occurs when the researcher is aware of the next treatment allocated for the patient, which often leads to selection bias in observational studies. Participants with IC who were referred for revascularisation or who were severely ischaemic were excluded from these studies, resulting in the recruitment of only those with milder disease. Subsequently the disease progression reported by a mean or percentage decline in ABPI in these studies, does not include those with more severe PAD and results therefore underestimate the true progression.

Survival selection bias was evident in 6 [24–29] out of the seven studies reviewed. This occurred when authors excluded participants who required revascularisation during the course of the study or who died by the end of the study. In the context of evaluating the natural history of PAD in IC patients, survival selection bias, sometimes referred to as selective reporting, often results in the underestimation of the true progression of the disease since those who most likely progressed rapidly were excluded from the analysis. Among the reviewed studies, this source of bias was often coupled with selection bias at recruitment stage, as discussed earlier, which may have exacerbated the possibility of underestimation of the progression of PAD in this group of patients.

The reporting of methodological detail about aspects that threaten internal validity such as measurement precision of the tools used were often reported. Having reliable and valid instruments is one of the best ways of reducing measurement bias in epidemiologic research. However, reports of measurement quality due to the possibility of arterial calcification and hence reporting, were also neglected, with only one article [26] reporting the possibility of artefactually elevated ABPI. While Walsh et al. [21] analysed atherosclerotic progression using sonographic studies, other studies which used the ABPI as a surrogate measure of peripheral perfusion, are susceptible to the hidden risks associated with this tool [31]. Patients with diabetes, smoking, renal disease or aged over seventy five are at a higher risk of medial arterial calcification [32, 33]. In these patients, the ABPI needs to be interpreted with caution since results may be artefactually elevated due to non-closure or delayed closure of the artery when the cuff is inflated. In such cases the TBPI is recommended since the digital arteries of the foot are less susceptible to arterial calcification [34, 35]. However, since most of these studies were published before issues with calcification and ABPI readings were recognized, the possibility of having artefactually elevated ABPI readings was not addressed. Among the studies included in this systematic review, only one study [26] reported TBPI readings while another six [24, 25, 27–30] did not discuss the possibility of falsely elevated results possibly resulting in an underestimation of the true decline of ABPI in their results [36].

## Discussion

This systematic review is the first to evaluate the progression rate of PAD in individuals with IC in terms of haemodynamic assessments of the lower limb, since previous reviews largely focused on mortality or amputation risks [37]. Data from the reviewed studies have generally agreed on an overall decline in ABPI by 0.01 to 0.02 over 1 year [24, 26, 28]. A decline of 0.15 was reported in severe cases of PAD [27] which has been previously associated with a 2.5 increased risk of surgical intervention [38] and is independently associated with increased risk of cardiovascular disease [39]. However, these results are probably an underestimation of the true overall deterioration in this population. The issues with selection bias at recruitment stage and the use of ABPI as a measure of peripheral perfusion with its inherent difficulties in the presence of medial arterial calcification [31, 40] probably resulted in a falsely conservative measure of PAD progression reported in most studies. Indeed, a faster progression rate was reported in only one study, stating an overall decrease of 0.14 in ABPI in 21% of patients within the first year [29].

The underestimated risk to the limb in PAD patients has also been reported in a systematic review investigating the progression of PAD in both asymptomatic and symptomatic patients within the context of amputation and mortality risk [37, 41]. The authors report that while the TransAtlantic Inter-Society Consensus for the Management of Peripheral Arterial Disease (TASC) state an amputation rate of 1–3% after five years [6] in IC patients, results from their review indicate a more aggressive progression of PAD resulting in an amputation rate of 27% in those with IC.

Current level of knowledge precludes the development of robust predictive formulae to identify the risk of haemodynamic deterioration in an individual patient. It is extremely likely that accurate prediction of patient specific risk of deterioration would lead to a paradigm shift in the management of patients with intermittent claudication. Delaying intervention until the patient has already developed critical ischaemia almost invariably means that more extensive occlusive disease has developed. The more complex and the more extensive the disease the more difficult and the riskier the intervention is and the lower the likelihood of success and long-term patency [30]. Furthermore, the incidence of MALE is associated with a very significantly increased risk of limb loss and death [12]. In a systematic review of treatment in IC [18], authors concluded that data related to the identification of the best suited intervention for each individual patient to achieve the most favourable outcome are still lacking. As a result of this uncertainly The Society for Vascular Surgery recommends that patients’ values and preferences should guide the clinical decision on intervention in patients with intermittent claudication [42]. Ideally however those preferences should be based on more robust evidence.

The poor design and reporting in the selected studies may have introduced bias and reduced the robustness of data [22, 43]. The studies evaluated in this systematic review have shown variable reporting of some of the major threats to the internal and external validity of observational longitudinal studies [22]. Selection bias due to inadequate allocation concealment, limited reporting of methodological detail, incomplete information related to attrition or non-consent and survival selection bias were the most common sources of bias observed in these studies. Despite ongoing efforts in research in this population, it is common in the literature for the main focus to be mortality and cardiovascular risk rather than the affected limb [37]. This trend has resulted in a limited understanding of the prognosis and progression rate of PAD in IC with no established criteria which allow the clinician to predict outcomes in an individual patient [44].

### Limitations of this review

The limitations of this review are mainly related to the significant heterogeneity of data among the studies due to different outcome measures and study cohorts. Therefore, meta-analyses of the data could not be performed and only descriptive analysis of the studies was presented. Methodological quality of the included studies was rigorously assessed.

Although efforts were made to carry out a thorough search of the literature, some studies may have been overlooked during the process.

### Future studies

This paper highlights the need for further research to evaluate the progression rate of PAD in patients with IC. Data from registries which include complete consecutive patient cohorts and are protected from selection bias, are important to support such knowledge. Future studies should conceal allocation and pursue a high rate of follow-up, with a maximum of twelve-month interval between reviews in order to capture any haemodynamic change and reduce the risk of survival selection bias observed in studies with long interval periods and high attrition rates. Due to the calcification risk and possibility of artefactually elevated ABPIs, future studies need to include Doppler waveform and toe-brachial pressure analysis since these assessment modalities are less susceptible to arterial calcification and more likely to provide reliable haemodynamic data.

## Conclusions

This review has shown that the existing knowledge on the natural progression of intermittent claudication is limited to a small number of studies providing mostly low-quality evidence related to measurable haemodynamic progression rate. The inherent difficulties associated with ABPI as a surrogate measure of peripheral perfusion in patients with medial arterial calcification and the probable underestimated rate of reported progression of PAD have been highlighted. Consequently, international guidelines on the management of PAD are necessarily generic. Further research into the natural progression of the disease is required to enable the development of predictive formulae to guide patient specific management of the condition.

## Data Availability

The datasets used and / or analysed during the current study are available from the corresponding author upon reasonable request.

## Abbreviations

CLI: Critical limb ischaemia
IC: Intermittent claudication
MALE: Major adverse limb event
PAD: Peripheral arterial disease,
PRISMA: Preferred Reporting Items for Systematic Reviews and Meta-Analyses
